# Transcriptome Analysis Revealed Long Non-Coding RNAs Associated with mRNAs in Sheep Thyroid Gland under Different Photoperiods

**DOI:** 10.3390/genes13040606

**Published:** 2022-03-28

**Authors:** Wei Wang, Xiaoyun He, Ran Di, Xiangyu Wang, Mingxing Chu

**Affiliations:** Key Laboratory of Animal Genetics, Breeding and Reproduction of Ministry of Agriculture and Rural Affairs, Institute of Animal Science, Chinese Academy of Agricultural Sciences, Beijing 100193, China; wangw8182@163.com (W.W.); hexiaoyun@caas.cn (X.H.); diran@caas.cn (R.D.); wangxiangyu@caas.cn (X.W.)

**Keywords:** sheep, thyroid gland, photoperiod, lncRNA, mRNA

## Abstract

The thyroid gland is a vital endocrine organ involved in the reproduction of animals via the regulation of hormone synthesis and secretion. LncRNAs have been proven to play important roles in reproductive regulation; however, the associated mechanism in the thyroid gland has not been clarified. In this study, we investigated to identify photoperiod-induced lncRNAs and mRNAs in the thyroid gland in Sunite ewes by comparing the expression profiles of short photoperiod (SP) and long photoperiods (LP). A total of 41,088 lncRNAs were identified in the thyroid gland through RNA-Seq. Functional analysis of differentially expressed lncRNAs using the R package revealed that reproductive hormone- and photoperiod response-related pathways, including the prolactin signaling, cAMP signaling, and circadian rhythm pathways, were significantly enriched. An mRNA-lncRNA interaction analysis suggested that the lncRNA *LOC1056153S88* trans targets *ARG2* and *CCNB3*, and the lncRNA *LOC105607004* trans targets *DMXL2*, both of these might be involved in seasonal sheep breeding reproduction. Together, these results will provide resources for further studies on seasonal reproduction in sheep.

## 1. Introduction

Animals have two kinds of reproduction mechanisms: seasonal reproduction and nonseasonal reproduction. Seasonal reproduction refers to the gonadal development and reproductive behavior of animals at specific times of the year, and it promotes the survival of the offspring. The midpoint of latitude is the main factor affecting the seasonality of reproduction, and it is related to photoperiod [1]. Studies have shown that most mammals have highly accurate endogenous photoperiod indicators, and small changes in photoperiod can cause a strong response from the body [2,3]. The pituitary pars tuberalis (PT) is a central target for melatonin that modulates seasonal functions, and it is considered the annual biological clock. In the PT, melatonin regulates the photoperiod by acting on thyroid-stimulating cells in the pituitary nodule, which in turn involves the transcriptional regulatory factor *EYA3* as an underlying mechanism [4,5]. The thyroid gland plays an important role in endogenously generated reproductive transitions in certain animals that present seasonal breeding. European starlings subjected to thyroidectomy before the breeding season, for example, do not experience the final transition of this reproductive season [6]. Recent evidence suggests that this phenomenon also exists in sheep mammals [7]. Specifically, ewes that had undergone thyroidectomy before the onset of the breeding season began to breed actively at a suitable time in the fall and continued to show estrus cycles during the subsequent nonbreeding season. These observations suggest that the presence of the thyroid is necessary for the endogenously induced transition out of the reproductive period during seasonal reproduction in certain species.

Long non-coding RNA (lncRNA) plays key roles in epigenetics and important regulatory roles in sheep reproduction. Studies have shown that there were differentially expressed lncRNAs in Hu sheep pituitary glands that are associated with high and low fecundity [8] and exert their role by regulating the expression of genes encoded by adjacent proteins in organisms. In recent years, many lncRNAs have been reported to be involved in the reproductive regulation of mice [9], rats [10], sheep [11,12], and goats [13]. LncRNAs can also significantly regulate the function of the thyroid gland, with some lncRNAs have been considered susceptibility markers of cancers, especially papillary thyroid cancer [14]. In addition, a few lncRNAs potentially associated with the pathogenesis of thyroid cancers [15,16].

Researchers currently have a limited understanding of how photoperiod induces the molecular neuroendocrine axis and reproductive seasonal changes. Studies on quails, hamsters, and sheep have mostly focused on hormone levels and compared the expression patterns of key genes or proteins in long photoperiod and short photoperiod [17,18]; however, a comprehensive understanding of photoperiod changes on the overall level of the thyroid gland transcriptome is lacking. The OVX model has been used to study the organ and tissue functions of mammals, such as rats, mice, goats, and sheep. In this study, the effects of photoperiod changes on the thyroid transcriptome were analyzed by high-throughput sequencing and bioinformatics methods based on our work [19]. The results of this study will provide some new information for understanding thyroid function.

## 2. Materials and Methods

### 2.1. Animal Treatments and Sample Collection

Based on the OVX+E2 model established by our team in a previous pre-experiment, nine Sunite ewes (2–3 years old) with the same body weight and nonpregnant were selected for OVX+E2 treatment [20]. The nine ewes were divided into three groups and were maintained in three rooms (Room 1: short photoperiod, SP, 8/16 h light-dark; Room 2: long photoperiod, LP, 16/8 h light-dark; and Room 3: short photoperiod transfer to long photoperiod, SP-LP) on a farm at the Tianjin Institute of Animal Sciences, Tianjin, China. All sheep were bred under the same diet and living conditions and provided food and water ad libitum. The thyroid glands of three ewes in each room were collected for SP 42 days, LP 42 days, and SP-LP42 days, frozen in liquid nitrogen and then stored at −80 °C for transcriptional sequencing analysis.

All the experimental procedures were authorized by the Science Research Department of the Institute of Animal Sciences, Chinese Academy of Agricultural Sciences (IAS-CAAS; Beijing, China). It is also ethically approved by the Animal Ethics Committee of the IAS (IAS2018-3).

### 2.2. RNA Extraction and Sequencing

Total RNA from the thyroid glands was isolated using the TRIzol reagent (Invitrogen, Carlsbad, CA, USA) according to the manufacturer’s instructions. The purity, concentration, and integrity of the RNA were determined by electrophoresis and a Bioanalyzer 2100 system, and RNA Nano 6000 Assay Kit (Agilent Technologies, Santa Clara, CA, USA). A RIN value above 7.5 indicates a qualifying sample that can proceed to the next step of the analysis. Subsequently, sequencing libraries of the nine samples were generated using the rRNA-depleted RNA with the NEBNext^®^ UltraTMDirectional RNA Library Prep Kit for Illumina^®^ (NEB, Ipswich, MA, USA). Finally, the libraries were sequenced on an Illumina platform.

### 2.3. Reference Genome Mapping and Transcriptome Assembly

Clean data were obtained by removing reads containing adapters and ploy-N and low-quality reads from raw data. Simultaneously, the Q30 of the clean data was calculated. The *Ovis aries* reference genome and gene annotation files were downloaded from the NCBI Genome website (Oar_v4.0) [21]. Paired-end clean reads of each sample were aligned by HISAT2 (v2.0.5) following the *Ovis aries* reference genome. The mapped reads of each sample were assembled by StringTie (v1.3.2.d).

### 2.4. LncRNA Identification and Differentially Expression Analysis

Potential lncRNA candidates had lengths that were two or more exons and transcripts longer than 200 nt. After calculating the expression of each transcription product by HTSeq (v0.6.0), the transcription product with FPKM ≥ 0.5 was maintained. Finally, CNCI [22], CPC [23], CPAT [24], and Pfam [25] were used to identify specific lncRNAs, and four junctions were adopted as the final candidate lncRNAs for subsequent analysis.

Three biological replicates were included in the experiment, and the FPKM value was used to estimate the expression levels of transcripts. The R package Ballgown was used to analyze differentially expressed transcripts after a negative binomial distribution. LncRNAs and mRNAs with *p* < 0.05 and log2 |Fold change| > 1 were considered as differentially expressed transcripts.

### 2.5. Functional Annotation and Enrichment Analysis

In a previous study, we had defined the cis and trans functions of lncRNAs [12]. GO, and KEGG enrichment analyses were performed using the R package [8]. According to the significant threshold (*q* < 0.05), it was considered to be significantly enriched compared to the GO and KEGG enrichment.

### 2.6. Construction of Integral lncRNA–mRNA Interaction Networks

To predict the function of DE-lncRNAs and their target genes in sheep reproduction, a network based on lncRNAs and mRNAs was established using Cytoscape (V3.8.2) [26].

### 2.7. Gene Expression Validation by Real-Time PCR

We selected nine mRNAs and nine random lncRNAs to validate the accuracy of RNA sequencing by the reverse-transcription quantitative polymerase chain reaction (RT-qPCR). β-actin was used as an internal reference to normalize target gene expression. Each qPCR experiment was performed 3 times, and the relative RNA expression value was calculated using the 2^−∆∆Ct^ method.

### 2.8. Statistical Analysis

All data were evaluated as the “means ± SD”. Experimental results were evaluated using Student’s *t*-test, and *p* < 0.05 was considered statistically significant.

## 3. Results

### 3.1. Summary of Sequencing Data in Thyroid Gland

Nine separate cDNA libraries were constructed from three groups (SP42, LP42, SP-LP42) of samples for high-throughput sequencing. As shown in Table 1, after removing low-quality sequences, each sample produced clean reads of more than 10.83 G, and the Q30 of the sample data was above 89%, which showed that the sequencing data were highly reliable (Table S1). In addition, more than 94.64% of each library’s clean reads and 94.32% of the total mapped reads were compared to the sheep reference genome (Oar_v4.0).

### 3.2. Differential Expression Analysis of lncRNAs and mRNAs

**Figure 1:**
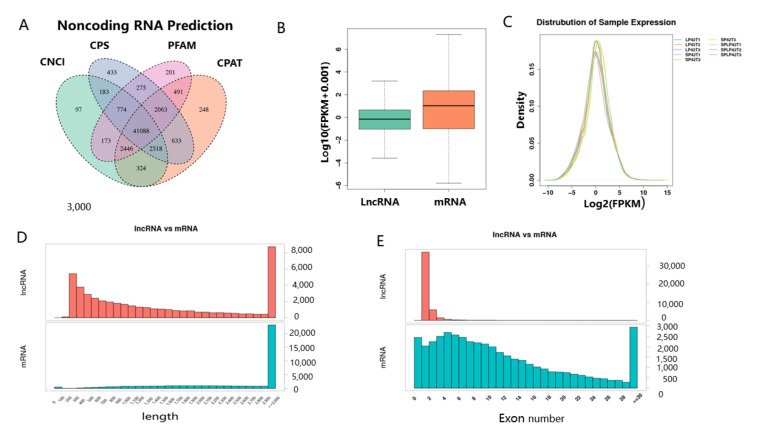
Identification of lncRNAs and mRNAs in the ewes thyroid gland. (**A**) Venn shows the common and unique number of novel lncRNAs by four methods, including CNCI, CPC, PFAM, and CPAT. (**B**) The expression level of lncRNAs and mRNAs. (**C**) FPKM distribution of each sample. (**D**) The length statistics of lncRNA and mRNA. (**E**) The statistics of lncRNA and mRNA exon number.

The results of four programs (CNCI, CPC, PFAM, and CAPT) were combined to select common novel lncRNAs. As a result, 41,088 novel lncRNAs were identified in the thyroid gland tissues of nine sheep (Figure 1A). It was clear that the transcriptional level of lncRNAs in the thyroid gland of Sunite ewes was lower than that of mRNAs (Figure 1B). The distribution trend of expression in nine samples was similar (Figure 1C). The lncRNAs length distribution was consistent with that of mRNAs (Figure 1D). The lncRNAs mainly contain two exons, which was significantly less than that of the mRNAs (Figure 1E).

### 3.3. Analysis and Verification of DE-lncRNAs and DE-mRNAs

**Figure 2:**
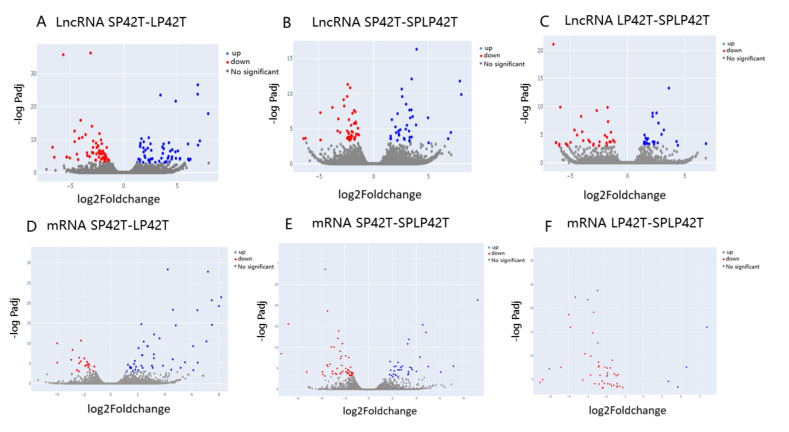
Analysis of differentially expressed transcripts. (**A**) Differentially expressed transcripts were assessed based on a volcano map of the DE-lncRNAs in SP42 and LP42. (**B**) Differentially expressed transcripts were assessed based on a volcano map of the DE-lncRNAs in SP42 and SPLP42. (**C**) Differentially expressed transcripts were assessed based on a volcano map of the DE-lncRNAs in LP42 and SPLP42. (**D**) Differentially expressed transcripts were assessed based on a volcano map of the DE-mRNAs in SP42 and LP42. (**E**) Differentially expressed transcripts were assessed based on a volcano map of the DE-mRNAs in SP42 and SPLP42. (**F**) Differentially expressed transcripts were assessed based on a volcano map of the DE-mRNAs in LP42 and SPLP42. Blue and red represent up-regulated and down-regulated transcripts, respectively (Table S2).

In total, 496 DE-lncRNAs and 280 DE-mRNAs were identified by comparing the LP42 to SPLP42 (Figure 2A,D), and 1078 DE-RNAs (including 727 DE-lncRNAs and 351 DE-mRNAs) were identified by comparing the SP42 to LP42, and 1068 DE-RNAs (including 664 DE-lncRNAs and 404 DE-mRNAs) were identified by comparing the SP42 to SPLP42.

### 3.4. GO and KEGG Enrichment Analysis of DE-mRNAs and DE-lncRNAs

**Figure 3:**
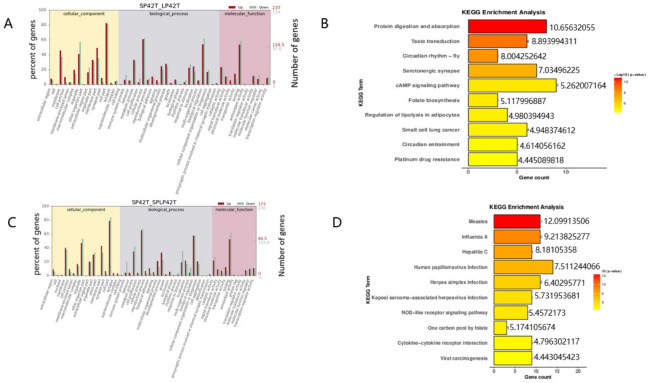
An analysis of GO and KEGG enrichment in the thyroid gland was performed. (**A**) An analysis of GO function of DE-mRNAs in SP42 and LP42. (**B**) Ten major KEGG enrichment pathways of DE-mRNAs were observed between SP42 and LP42. (**C**) An analysis of GO function of DE-mRNAs in SP42 and SPLP42. (**D**) Ten major KEGG enrichment pathways of DE-mRNAs were observed betweenSP42 and SPLP42.

**Figure 4:**
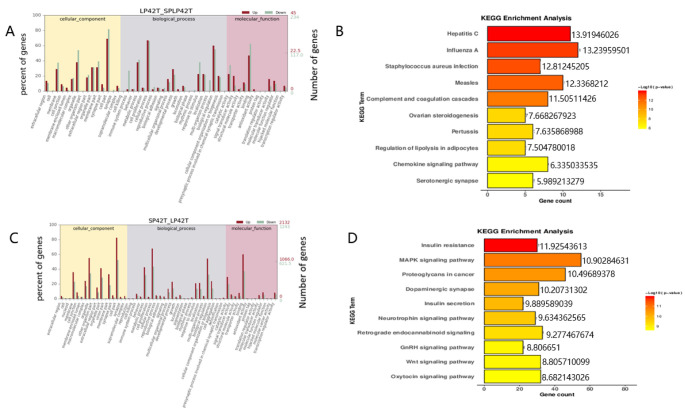
An analysis of GO and KEGG enrichment in the thyroid gland was performed. (**A**) Analysis of GO function of DE-mRNAs in LP42 and SPLP42. (**B**) Ten major KEGG enrichment pathways of DE-mRNAs were observed between LP42 and SPLP42. (**C**) Analysis of GO function of DE-lncRNAs in SP42 and LP42. (**D**) Ten major KEGG enrichment pathways of DE-lncRNAs were observed between SP42 and LP42.

**Figure 5:**
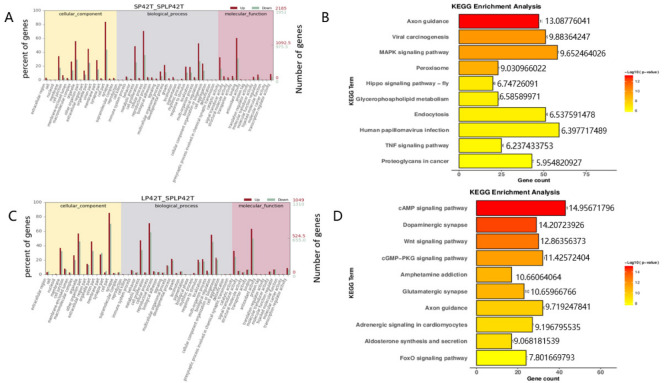
An analysis of GO and KEGG enrichment in the thyroid gland was performed. (**A**) Analysis of GO function of DE-lncRNAs in SP42 and SPLP42. (**B**) Ten major KEGG enrichment pathways of DE-lncRNAs were observed between SP42 and SPLP42. (**C**) Analysis of GO function of DE-lncRNAs in LP42 and SPLP42. (**D**) Ten major KEGG enrichment pathways of DE-lncRNAs were observed between LP42 and SPLP42.

GO and KEGG analyses were performed for the target genes of differentially expressed mRNAs and differentially expressed lncRNAs. GO and KEGG enrichment analysis results of the DE-mRNAs and DE-lncRNAs in the thyroid gland were shown in Figure 3, Figure 4 and Figure 5 (Tables S3 and S4). Most significantly, For the DE-mRNAs and DE-lncRNAs, the top 10 enriched terms of each GO type and the top 10 major enrichment pathways are reported.

A number of GO terms are noteworthy, and they included neuron terms (such as neuron part, axon, synapse part, and response to stimulus), receptor terms (such as cell surface receptor signaling pathway and receptor complex), regulation of apoptosis process, regulation of cell death, regulation of cell proliferation, and metabolic process (such as regulation of metabolic process and positive regulation of phosphorus metabolic process).

The KEGG enrichment analysis of DE-mRNAs identified several enriched pathways. The most enriched pathway between SP42 and LP42 was the cAMP signaling pathway, Although the circadian rhythm-fly associated with circadian rhythm was also enriched (Figure 3B). The most enriched pathway SP42 and SPLP42 was Human papillomavirus infection, and pathways associated with the disease were also enriched (Figure 3D). In addition, many genes that were differentially expressed between LP42 and SPLP42 were enriched in reproduction-related pathways, such as signaling (prolactin signaling pathway and cAMP signaling pathway), neurons, neurotransmitters, and cell processes (Figure 4B). The KEGG enrichment analysis of DE-lncRNAs also identified several enriched pathways. The most interesting pathways between SP42 and LP42 included reproduction-related pathways (Oxytocin signaling pathway, Wnt signaling pathway, MAPK signaling pathway, and GnRH signaling pathway, as shown in Figure 4D). The results showed that many genes differentially expressed between SP42 and SPLP42 were enriched in reproduction-related pathways, such as the MAPK signaling pathway and TNF signaling pathway (Figure 5B). Figure 5D also showed that many genes differentially expressed between LP42 and SPLP42 were enriched in pathways related to reproduction, such as the Wnt signaling pathway and cAMP signaling pathway.

### 3.5. LncRNA–mRNA Network Construction

**Figure 6:**
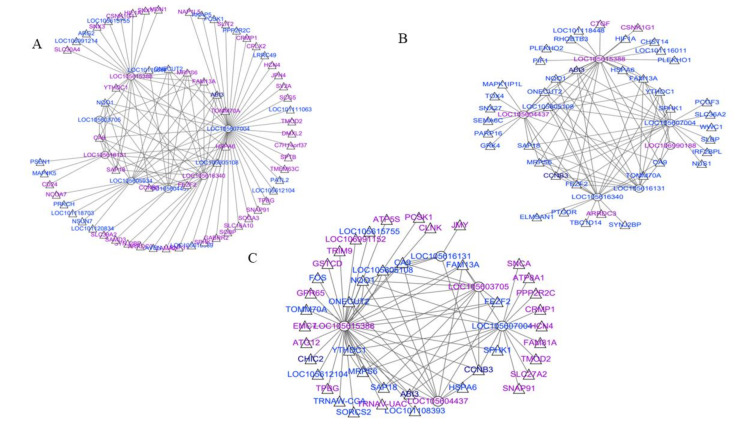
The interaction networks were identified for lncRNAs and their corresponding target genes. (**A**) The networks for SP42 and LP42; (**B**) the networks for SP42 and SPLP42; (**C**) the networks for LP42 and SPLP42. Blue and purple represent up and downregulation, respectively. Triangle and Ellipse represent mRNAs and lncRNAs, respectively.

To investigate the molecular mechanism underlying the effect of the illumination time on sheep estrus, we constructed a co-expression network based on the expression levels of DE-lncRNAs and DE-mRNAs (Table S5). In the SP42 and LP42 groups, 10 known lncRNAs corresponded to 67 target genes. Notably, *LOC105615388* was also found to trans-target *ARG2,* while *CCNB3* and *LOC105607004* were found to *DMXL2* because both genes are important for seasonal reproduction (Figure 6A). The network of SP42 and SPLP42 contained six differentially expressed lncRNAs and 42 target genes, and a trans relationship between *LOC105604437* and *SEMA6C* has been proposed (Figure 6B). In LP42 and SPLP42, five differentially expressed lncRNAs and 43 target genes were selected to construct the network. For some core genes associated with seasonal breeding, several target combinations between lncRNAs and these genes were demonstrated. For example, they suggested that *LOC105615388* likely trans-regulated *SAP18*, *LOC105607004* likely trans-regulated *CCNB3* (Figure 6C).

### 3.6. Gene Expression Validation

**Figure 7:**
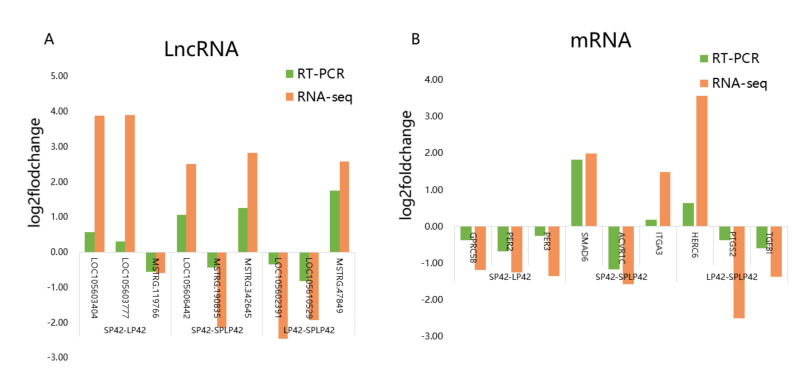
Validation of RNA sequencing (RNA-seq) data using RT-qPCR. (**A**) RNA-Seq and RT-qPCR results of three selected differentially expressed lncRNAs in the thyroid gland of Sunite sheep at different photoperiods. (**B**) RNA-Seq and RT-qPCR results of three selected differentially expressed mRNAs in the thyroid gland of Sunite sheep at different photoperiods.

A total of 18 genes, namely, including nine mRNAs (*GPRC5B*, *PER2*, *PER3*, *SMAD6*, *ACVR1C*, *ITGA3*, *HERC6*, *PTGS2,* and *TGFBI*) related to seasonal reproduction and nine random lncRNAs (*LOC105603404*, *LOC105603777*, *MSTRG.119766*, *LOC105606442*, *MSTRG.190835*, *MSTRG.342645*, *LOC105602391*, *LOC105610529*, and *MSTRG.47849*), were selected for RT-qPCR verification. The results indicated that there was a similar expression pattern between RNA-Seq and RT-qPCR data (Figure 7, Table S6).

## 4. Discussion

Seasonal adaptations of animal reproductive maximize the survival of offspring. However, the molecular mechanism underlying the seasonal breeding of animals has not been clarified in detail. So far, it has been known for many decades that thyroid hormones have been known to regulate reproductive function in both birds and mammals [27]. The thyroid gland is an important regulator of seasonal reproductive traits, and increasing evidence has shown that seasonal reproduction is regulated by the hypothalamic-pituitary-thyroid (HPT) axis. TRH (thyrotropin-releasing hormone) secreted from the hypothalamus in the HPT axis induces the pituitary to release thyroid-stimulating hormone (TSH), which in turn stimulates the thyroid gland to synthesize and release TH [3,28]. The study has confirmed that local activation of thyroid hormone metabolism is central to the regulation of the photoperiodic response has been confirmed in rats [27], mice [29], goats [30], and sheep [31] et al. Therefore, the primary purpose of this study, is to screen new photoperiod-induced candidate genes in ovine thyroid glands, which is the target tissue.

Both GO and KEGG pathway enrichment analyses are differentially expressed RNAs that were associated with pathways various signaling pathways, including the prolactin signaling pathway, cAMP signaling pathway, oxytocin signaling pathway, MAPK signaling pathway, GnRH signaling pathway, TNF signaling pathway, and Wnt signaling pathway. Growing evidence has shown that a temporary decrease in cAMP levels initiates downstream pathways, including diploid cessation to spontaneous meiosis resumption and induction of oocyte maturation [32]. Seasonal reproduction is known to be controlled by the hypothalamic-pituitary-gonad (HPG) axis. On the HPG axis, GnRH is secreted by the hypothalamus and stimulates the release of luteinizing hormone and follicle-stimulating hormone. These hormones act on the gonads, thereby promoting the development of these organs and the production of steroid hormones. *Chir98014* can act as a pathway activator of the Wnt/β-catenin signaling pathway to affect in oocyte in vitro maturation of Sanjabi ewes and subsequent embryonic development [33]. The MAPK signaling pathway plays a critical role in cell proliferation, differentiation, and cell migration. Thus, the prolactin signaling pathway, cAMP signaling pathway, oxytocin signaling pathway, Wnt signaling pathway, MAPK signaling pathway, GnRH signaling pathway, TNF signaling pathway, and Wnt signaling pathway are very important for seasonal reproduction. Several important candidate genes, including *CCNB3*, *SEMA6C*, *SAP18,* and *DMXL2,* were knocked out. More interestingly, *PER3* and *PER2* were shown to be enriched in the photoperiodic change-related pathway circadian entrainment. *CCNB3* has been reported to play an important regulatory role in MII arrest and exit in mouse oocytes [34]. Our results of the thyroid gland of ewes also further confirm that *CCNB3* is highly expressed under SP, which affects animal reproductive activities. A previous study showed that *SEMA6C* is involved in the activation of primitive follicles, and downregulation of *SEMA6C* expression leads to large-scale activation of primitive follicles, which has a huge impact on animal reproductive activities [35]. In our study, the photoperiod also modulated the expression of the *SEMA6C* gene and maintained high levels of the expression in the thyroid gland of hamsters during SP compared to LP. Moreover, *SAP18* has been shown to participate in epigenetic and transcriptional regulation, and its expression of *SAP18* in early gonadal development is significantly different between sexes, which helps clarify the genetic mechanism of gonadal development. Investigations in a *DMXL2*-deficient mouse line showed that haploid deficiency of *DMXL2* in neurons causes infertility due to a partial GnRH deficiency [36,37]. *DMXL2* can be regarded as a new candidate photoperiod-induced genes in the thyroid gland of sheep. Various PDE inhibitors elevate cyclic nucleotides levels reversibly inhibiting spontaneous exit from diplotene arrest, oocyte maturation, ovulation, and pregnancy [32]. *PER3* and *PER2* are typical clock genes whose expression is significantly correlated with circadian rhythm [38]. Our study showed that photoperiod can alter the reproductive status of animals through transcriptional regulation of key pathways at the mRNA level. This finding was consistent with previous results.

LncRNAs, as one of the most common ncRNAs, have attracted increasing attention from researchers because they participate in different ways in the regulation of various controlling biological processes. Studies have shown that lncRNAs play important roles in many aspects of reproductive regulation in sheep, such as fecundity [19]. This study found that changes in lncRNAs were involved in the seasonal reproduction regulatory mechanisms and identified many differentially expressed lncRNAs. Thus, another objective of this study was to predict lncRNAs that target core genes for seasonal reproduction in the thyroid gland of sheep. Firstly, our results showed that the length and exon count for mRNAs and lncRNAs sequence are different from those in the hypothalamus of sheep (3448 nt and 2.5 exons) [12]. This finding shows that lncRNAs are tissue-specific. GO and KEGG pathway analyses predicted that these differentially expressed lncRNAs were functionally linked to hormonal regulation and metabolism-related pathways. More importantly, significantly differentially expressed lncRNAs targeted at significantly differentially expressed mRNAs, which were associated with the protein metabolic process, secretion regulation, and developmental process. For example, *LOC105615388* was also found to trans-regulate *ARG2*, *CCNB3,* and *LOC105607004* trans-regulated *DMXL2*. Moreover, the established network revealed the trans relationship between *LOC105604437* and *SEMA6C*, *LOC105607004* and *CCNB3*, *LOC105615388* and *SAP18*, and *LOC105607004* and *DMXL2*. Notably, the trans relationship between *LOC105615388* and *CCNB3* was the same in all three comparison groups, indicating that *LOC105615388* might play an important role in seasonal reproduction by regulating the expression of *CCNB3*. As suggested, this study provides a new molecular object (*LOC105615388*) for the follow-up epigenetic regulation study of seasonal reproduction in sheep. In addition, candidate lncRNAs mentioned above (e.g., *LOC105604437* and *LOC105607004*) also require further analysis. The DE-lncRNAs identified from these data are presumed to play an essential role in the seasonal reproduction of sheep.

## 5. Conclusions

This study provided a gene transcriptional regulatory network of lncRNA and mRNA expression profiling in the ovine thyroid gland during LP and SP, and several new candidate photoperiod-induced genes and lncRNAs targeting key genes of seasonal reproduction were screened in the thyroid gland of sheep. These results will provide a valuable resource for understanding the molecular regulation of sheep prolificacy.

## Figures and Tables

**Table 1:** Summary of raw reads after quality control and mapping to the reference genome.

Sample Name	Raw Reads	Clean Reads	Clean Reads Rate (%)	Clean Bases (G)	Q30 (%)	Total Mapping Rate (%)
LP42Ta	125,086,648	120,862,882	96.62	18.13	93.95	95.43
LP42Tb	114,464,214	110,619,664	96.64	16.59	94.15	95.11
LP42Tc	127,822,204	122,122,532	95.54	18.32	93.76	94.39
SP42Ta	128,205,226	123,710,152	96.49	18.56	94.17	94.32
SP42Tb	124,015,570	120,265,994	96.98	18.04	94.35	95.22
SP42Tc	121,918,860	115,388,502	94.64	17.31	94.05	94.69
SPLP42Ta	113,613,174	108,371,314	95.39	16.26	90.27	95.27
SPLP42Tb	132,383,336	127,709,202	96.47	19.16	89.88	95.09
SPLP42Tc	132,355,318	128,440,756	97.04	19.27	89.23	95.84

LP42Ta represents thyroid gland (T) sample “a” in SP42.

## Data Availability

Not applicable.

## Supplementary Materials

The following supporting information can be downloaded at: https://www.mdpi.com/article/10.3390/genes13040606/s1, Table S1: Summary of raw reads; Table S2: Volcano map of differentially expressed lncRNA and mRNA; Table S3: Enriched GO and KEGG terms of DE-mRNAs; Table S4: Enriched GO and KEGG terms of DE-lincRNAs; Table S5: The data of betweenness, network degree; Table S6: The data of RNA sequencing.
